# Global prevalence of musculoskeletal disorders among physiotherapists: a systematic review and meta-analysis

**DOI:** 10.1186/s12891-023-06345-6

**Published:** 2023-04-04

**Authors:** Philippe Gorce, Julien Jacquier-Bret

**Affiliations:** 1 International Institute of Biomechanics and Occupational Ergonomics, Toulon, France; 2 Université de Toulon, CS60584-83041 - TOULON CEDEX 9, Toulon, France; 3 Hôpital Léon Bérard, Avenue du Docteur Marcel Armanet, Hyères, 83418 France

**Keywords:** Musculoskeletal disorders, Prevalence, Body area, Physiotherapists, Meta-analysis, Meta-regression, Systematic review

## Abstract

**Background:**

Musculoskeletal disorders (MSD) are one of the most important problems among physiotherapists worldwide. However, there is no meta-analysis of the MSD prevalence in all body areas among physiotherapists.

**Objectives:**

The purpose was to investigate and estimate the worldwide prevalence of MSD among physiotherapists using a systematic review-, meta-analysis and meta-regression.

**Methods:**

The systematic review, meta-analysis and meta-regression were performed in 2022 using the PRISMA guidelines.

**Data sources:**

The search was performed on PubMed/Medline, ScienceDirect, Google Scholar, Medeley and Science.gov databases.

**Study appraisal:**

The quality appraisal of the included articles was assessed using the critical appraisal tool for cross-sectional studies AXIS.

**Results:**

A total of 722 articles were found. After screening and comparison with the inclusion criteria, 26 studies were retained. Based on the random-effects model, the worldwide MSD prevalence in neck, upper back, mid back, lower back, shoulders, elbows, wrists/hands, thumb, hips/thighs, knees/legs, and ankles/feet was 26.4% (CI 95%: 21.0–31.9%), 17.7% (CI 95%: 13.2–22.2%), 14.9% (CI 95%: 7.7–22.1%), 40.1% (CI 95%: 32.2–48.0%), 20.8% (CI 95%: 16.5–25.1), 7.0% (CI 95%: 5.2–8.9), 18.1% (CI 95%: 14.7–21.5%), 35.4% (CI 95%: 23.0–47.8), 7.0% (CI 95%: 5.2–8.8), 13.0% (CI 95%: 10.3–15.8), and 5% (CI 95%: 4.0–6.9) respectively. The neck and shoulder prevalence of four continents were close to the world prevalence. No effect of continent was found on MSD prevalence. The heterogeneity of the results obtained in the meta-analysis and meta-regression was discussed.

**Conclusions:**

Based on the random effects model, the results of the worldwide meta-analysis showed that lower back pain, thumb, neck and shoulder were the area most at risk for MSD and were therefore those to be monitored as a priority. Recommendations were proposed for future reviews and meta-analyses.

## Introduction

Musculoskeletal disorders (MSD) in the occupational environment are a major public health issue in the world. They are responsible for multiple work stoppages and significant direct and indirect costs [1]. They affect both work habits and quality of work, quality of life and well-being of workers. Health professionals are populations at risk due to their varied interventions requiring significant loads [2–4].

In this context, the practices of physiotherapists (PT) expose them significantly to MSD. Indeed, several risk factors have been identified inducing important physical loads. Bending and twisting the trunk, transferring patients, performing manual therapy, working in awkward postures for long periods of time or repeating the same movements over and over are all factors that contribute and reinforce the presence of MSD and associated symptoms [5–7].

Many studies have addressed the high prevalence of MSD among PT worldwide. In studies by Khairy et al. [8], Kinaci et al. [9] or Grooten et al. [10], prevalence rates exceeded 80%. The 90% threshold has been reported in some countries such as Korea, Australia or the USA [2, 5, 11]. Several studies have investigated the prevalence of MSD by body area. Some work focused on certain areas such as the thumb [12, 13] or the upper limb [14, 15]. Other work extended the analysis to the lower limbs by including MSD hazards in the hip, knee and ankle [6, 11, 16]. Vieira et al. [17] provided an overview of MSD among physical therapists by summarizing the prevalence by body area during the career of PT. Ten areas were reported among the 32 included studies. The authors reported that lower back was the body area most commonly affected by MSD.

However, two limitations can be addressed. The first concerns the heterogeneity of the reported results. The prevalence rates could be based on two different samples. Some studies presented WMSD prevalence in relation to the whole sample tested [18], whereas other works reported prevalence rates in relation to participants who mentioned the presence of WMSD [9]. In this case, the rates were increased since people without WMSD were not considered. It therefore appears important to normalize these data to have an accurate estimation of the MSD prevalence in PT. The objective of this study was to perform a systematic review, meta-analysis and meta-regression of the MSD risk in PT normalized to the tested samples. The results would provide an assessment of MSD risk by body area by considering the prevalence of the different works conducted worldwide. The effect of the continent on the prevalence of MSD was also tested.

## Methods

The present systematic review and meta-analysis were conducted on the prevalence of MSD among physiotherapists in 2022. The study was reported according to Preferred Reporting Systematic Reviews and Meta-Analyses (PRISMA) guidelines [19]. The protocol for this review was registered at PROSPERO (CRD42023343473).

### Search strategy and eligibility criteria

Five international databases namely PubMed/Medline, ScienceDirect, Google Scholar, Medeley and Science.gov were explored in March 2022. The following keywords were used: (“Physiotherapy” OR “Physiotherapist” OR “Physical therapy” OR “Physical therapist”) AND “Musculoskeletal disorders” AND “Prevalence” AND “Body area”. The search focused exclusively on English language peer-reviewed works that quantified the MSD prevalence by body area among physiotherapists. Reviews, systematic reviews, commentaries, case studies and case series were not retained. Studies were excluded if: not published in English, not among physiotherapists, no sufficient data about sampling, the number of body parts is too low, mixed healthcare professions without the possibility of distinguishing them, or insufficient MSD prevalence details.

**Fig. 1:**
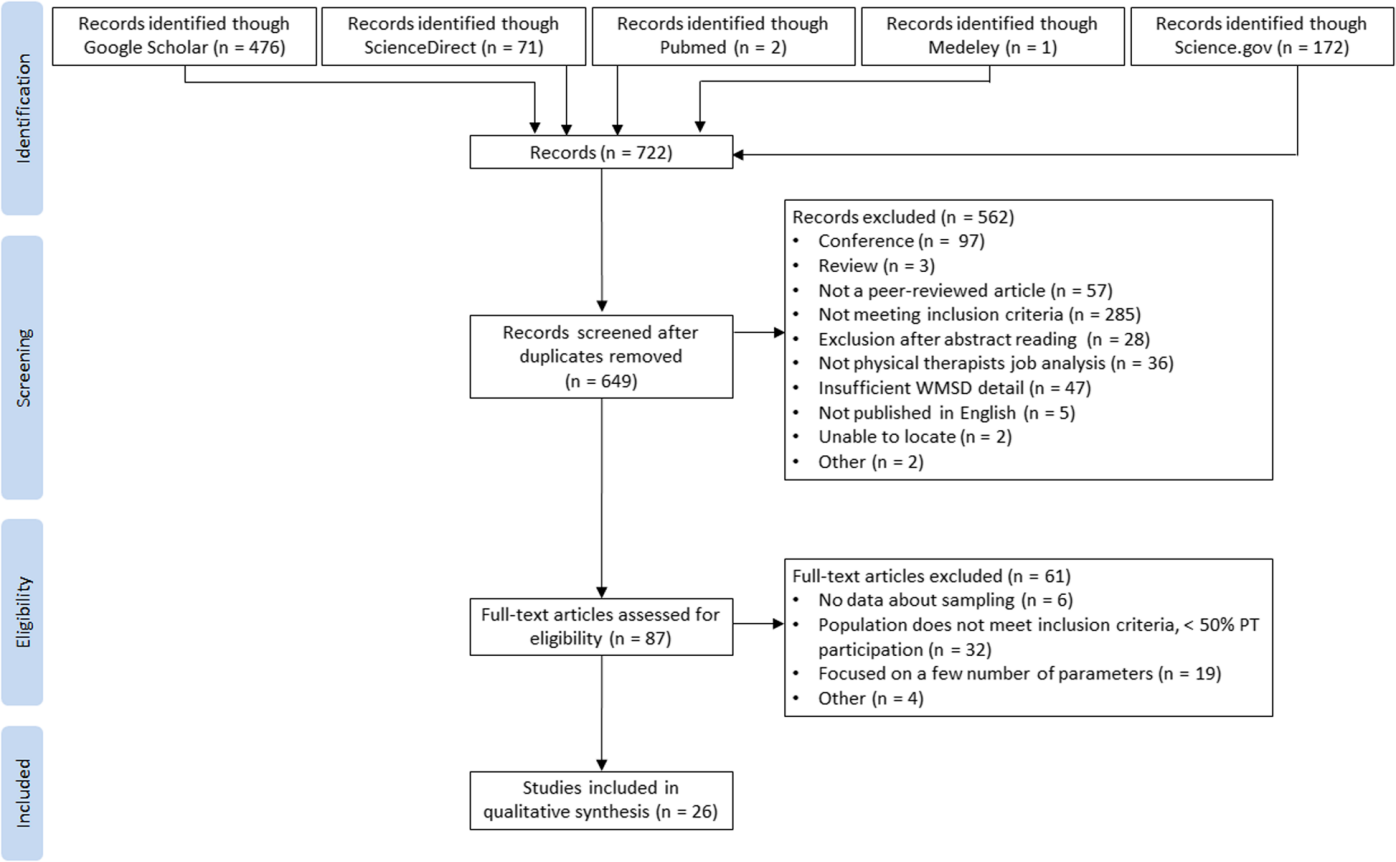
PRISMA Flow Chart

Results were imported from the five databases and compiled to remove duplicates. Titles and abstracts of unique records were separately screened by two reviewers (P.G. and J.J.B.) for eligibility. The full text of each article selected from its title/abstract was evaluated on the basis of the inclusion criteria separately by two reviewers. Studies that did not meet the criteria were excluded. All discrepancies were resolved by consensus and re-review of the articles. The search process is shown in Fig. 1.

### Methodological quality assessment and risk of bias

The critical appraisal tool was used to assess the quality of cross-sectional studies (AXIS) included in the review [20].

Each of the criteria was evaluated on its presence (“Yes”) or absence (“No”). The percentage of items present is then calculated. The quality appraisal was obtained using McFarland et al. [21] classification and the AXIS repartition: 0–50% has high risk of bias, 50–80% has medium risk of bias and 80–100% has low risk of bias. Two reviewers (P.G. and J.J.B.) performed the quality assessment separately. The discrepancies have been discussed for the final evaluation, involving a third reviewer where necessary.

### Data extraction

The included articles were used to extract the following data: number of respondents, response rate, male and female repartition, age, country, and MSD prevalence by body area. When prevalence rates were calculated from the subsample of physiotherapists with MSD, they were reported to the total sample tested to perform the meta-analysis with homogeneous data.

### Statistical analysis

The meta-analysis was performed based on the work of Neyeloff et al. [22]. Heterogeneity of the studies was assessed using Cochran's Q test (significance level < 10%) and I^2^ statistic (significance level > 50%). In case of heterogeneity, random effects model with inverse-variance approach was employed. Otherwise, fixed effects model was applied. A Kurskal-Wallis test was used to compare the prevalence of each body area on the five continents (significance level set at 5%). A meta-regression was performed to analyze the trend in MSD prevalence as a function of the average age of the participants, the year of publication of the included studies, and the Gross Domestic Product (GDP) of the country in which the study was conducted. Analyses were achieved using Statistica (Version 7.1, Statsoft, Tulsa, OK, USA).

## Results

### Search results

The exploration of the various databases identified 722 articles. Of the 649 unique articles, 87 articles were selected on the basis of their title/abstract and were fully evaluated. After comparison with the inclusion/exclusion criteria, 61 were excluded because either the data were mixed and did not meet the objective or the parameters studied were insufficient. Finally, 26 articles were retained and included in the analysis.

### Quality appraisal

The quality appraisal of the 26 included articles revealed that 18 studies had low risk of bias whereas 8 had medium risk of bias (Table 1.).

**Table 1.**
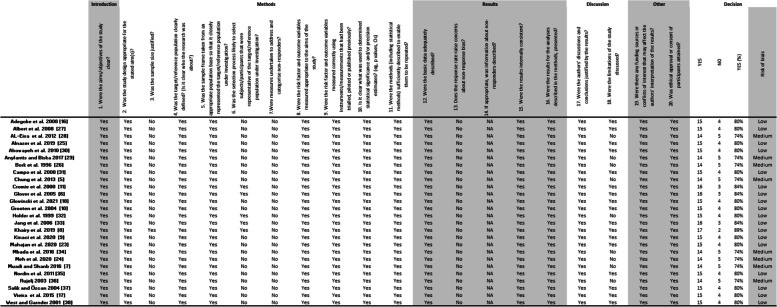
Detailed quality appraisal for risk of biais according to the AXIS [20]

### Study characteristics

All studies included in the review were surveys of MSD risk by body area among physiotherapists, physical therapists, massage therapists and kinesitherapists. The studies were conducted in 17 different countries on the 5 continents. The sample sizes were very heterogeneous ranging from a subgroup of 37 participants [18] to 2688 [6]. The response rate was also highly variable between studies, ranging from 37% [9, 23] to 91.9% [24]. All participants were adult men and/or women between 18 and 55 years old (mean were between 24.25 ± 7.27 years [25] and 43.0 ± 12.0 years [26]). Except the study conducted by Grooten et al. [10] that included only women, all other studies were performed with mixed population but with varying proportions.

Table 2. summarizes the general population characteristics, i.e. number of participants, response rate, men/women repartition, mean age, country, and the prevalence of MSD by body area of the 26 included studies. Eleven areas were assessed. The most studied were neck and lower back mentioned in all the 26 studies. Shoulder and wrist/hand were studied in 24 studies. Upper back, elbow/forearm, hip/thigh, knee/leg, and ankle/foot were addressed in 21 studies. Finally, thumb and mid back were the less studied body areas addressed in 7 and 3 studies respectively.

**Table 2.**
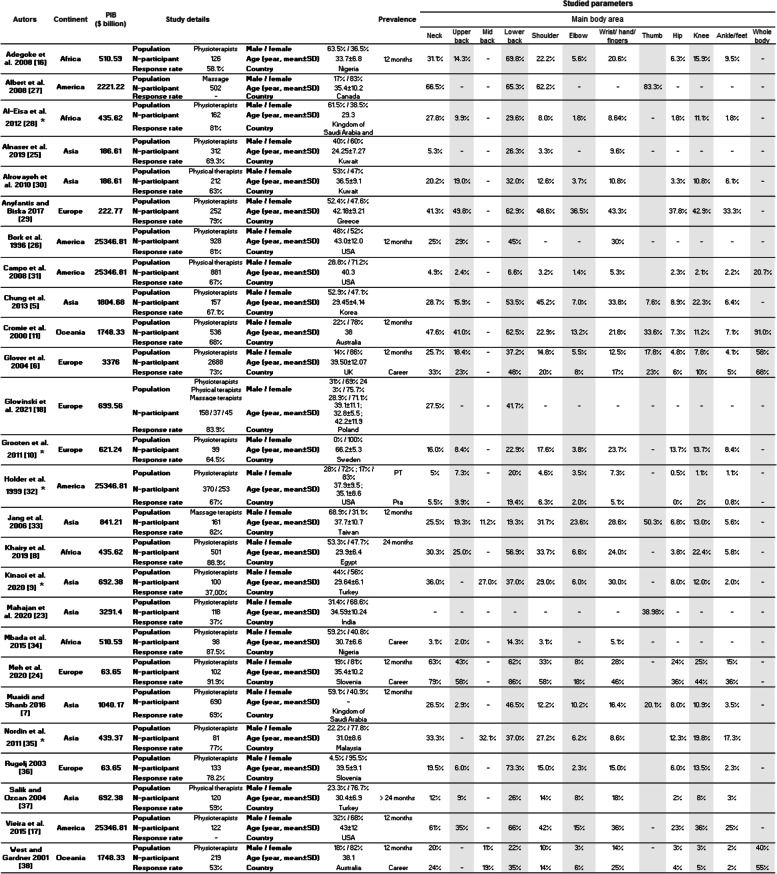
Objective and characteristics of the 36 included studies by healthcare profession. MSD prevalence by body area was reported for each study (when available)

### Meta-analysis results

Heterogeneity among studies was assessed using Q and I^2^ statistics. Results revealed important heterogeneity for all body areas: neck (Q = 1149.4; df = 25; I^2^ = 97.8%; *p* < 0.001), upper back (Q = 888.5; df = 20; I^2^ = 97.7%; *p* < 0.001), mid back (Q = 8.47;df = 2; I^2^ = 76.4; *p* < 0.05), lower back (Q = 1299; df = 25; I^2^ = 98.1%; *p* < 0.001), shoulder (Q = 828.3; df = 23; I^2^ = 97.2%; *p* < 0.001), elbow/forearm (Q = 248.4; df = 20;

I^2^ = 91.9%; *p* < 0.001), wrist/hand/finger (Q = 434.8; df = 23; I^2^ = 94.7%; *p* < 0.001), thumb (Q = 349.2; df = 6; I^2^ = 98.3%; *p* < 0.001), hip/thigh (Q = 371.2; df = 20;

I^2^ = 94.6%; *p* < 0.001), knee/leg (Q = 446.3; df = 20; I^2^ = 95.5%; *p* < 0.001), ankle/foot.

#### Neck

**Fig. 2:**
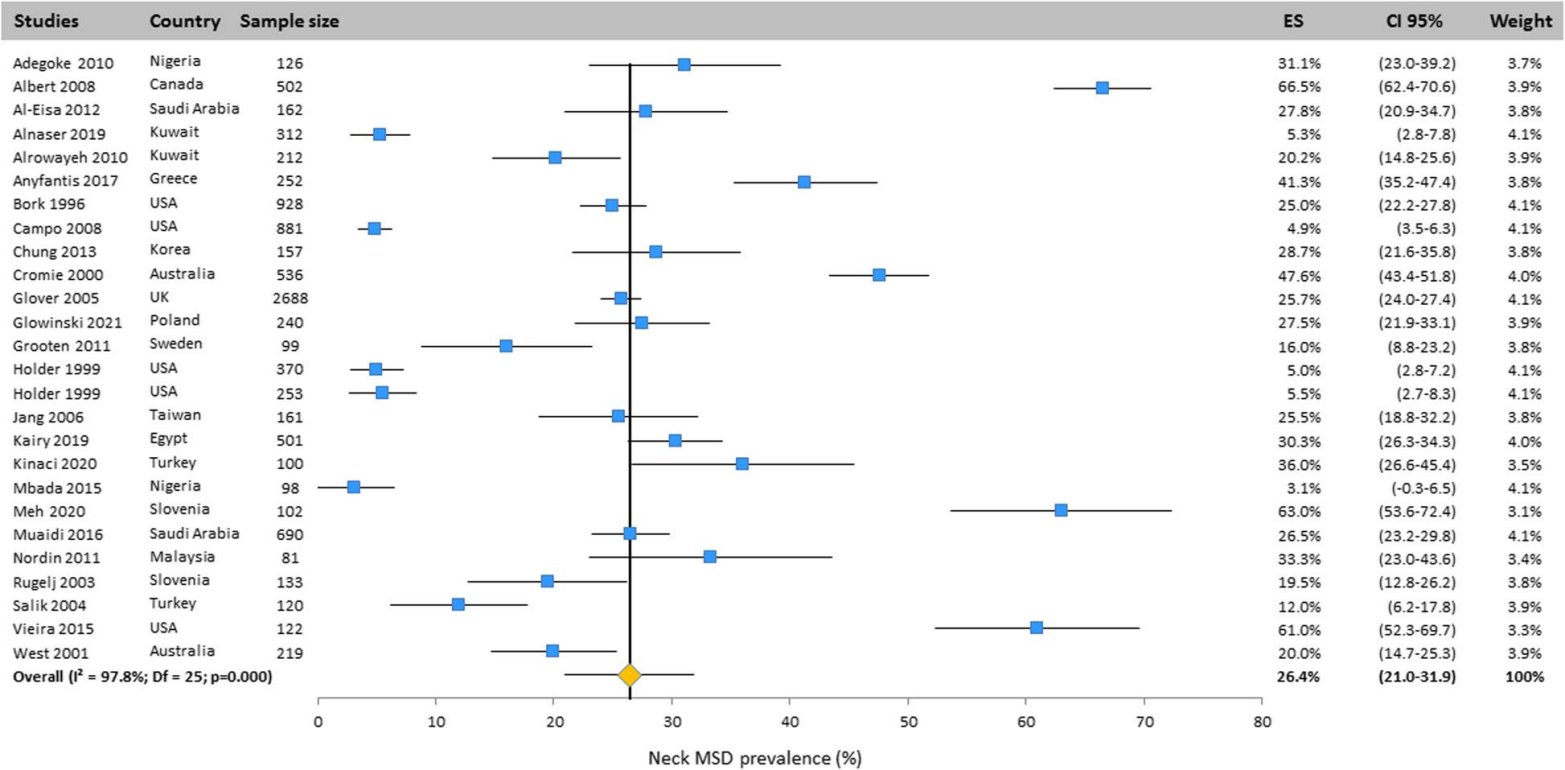
Prevalence of musculoskeletal disorders in neck amongst studies included

The prevalence of MSD for the neck was presented in all included studies (26 studies) carried out in many countries of the world (Fig. 2). Based on the random effects model, the neck prevalence was 26.4% (CI 95%: 21.0–31.9%).

#### Upper back

**Fig. 3:**
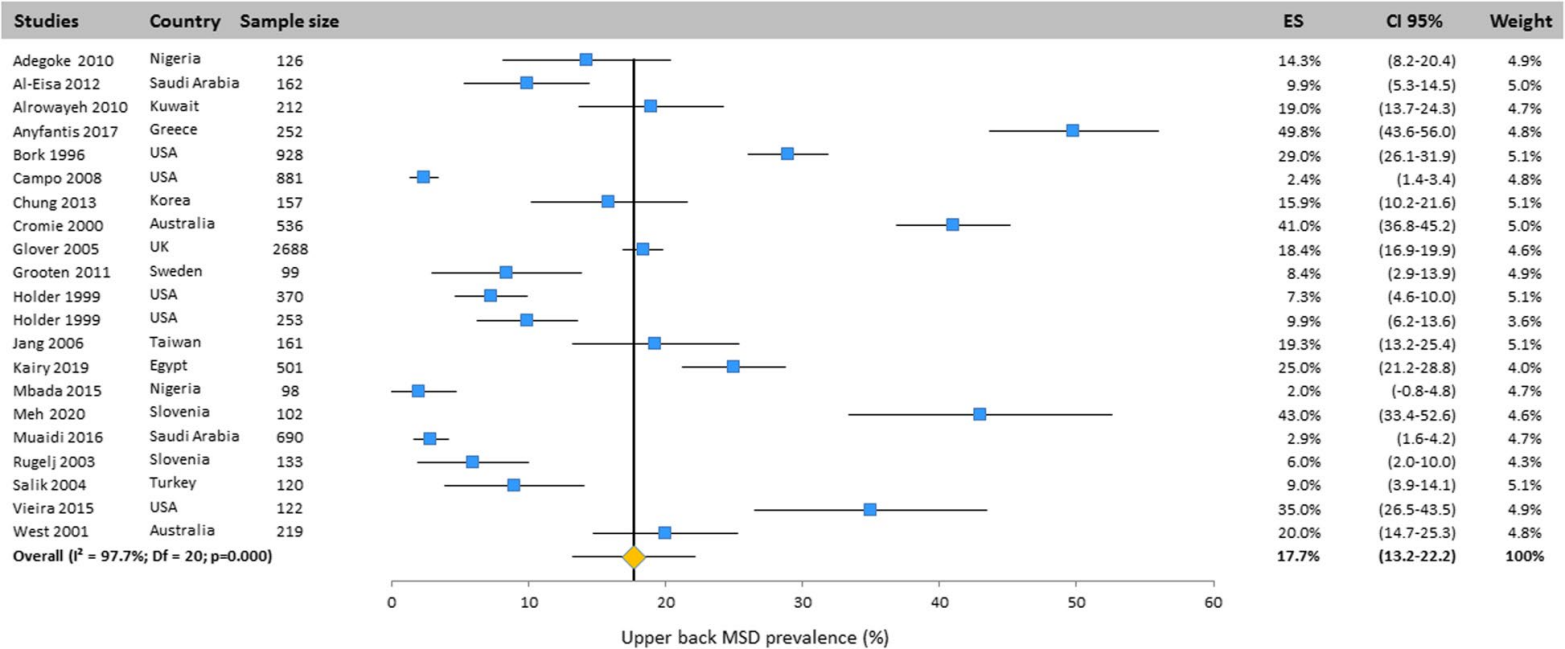
Prevalence of musculoskeletal disorders in upper back amongst studies included

The upper back MSD prevalence was evaluated in 21 studies around the world. The overall prevalence was 17.7% (CI 95%: 13.2–22.2%) obtained with the random effects model (Fig. 3).

#### Mid back

**Fig. 4:**
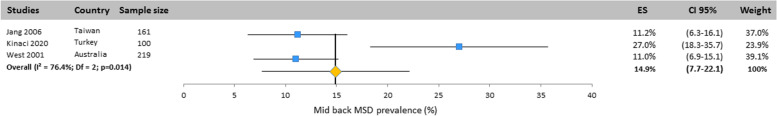
Prevalence of musculoskeletal disorders in mid back amongst studies included

The prevalence of MSD in the mid back has been the least studied, with only three studies reporting results (Fig. 4). These data came from Taiwan, Turkey, and Australia. The random effects model estimates the prevalence of mid back MSD at 14.9% (CI 95%: 7.7–22.1%).

#### Lower back

**Fig. 5:**
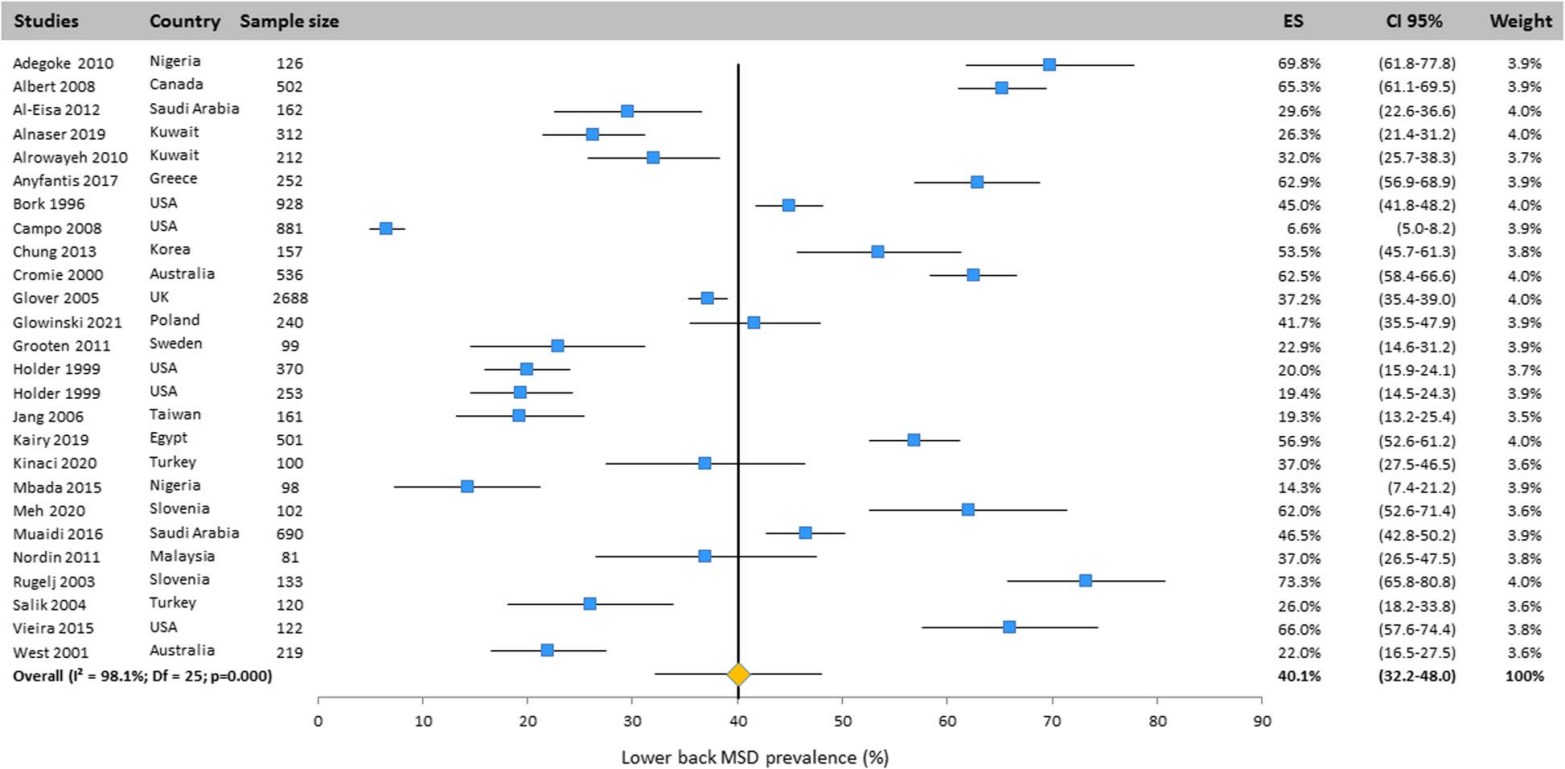
Prevalence of musculoskeletal disorders in lower back amongst studies included

As for the neck, the prevalence of lower back MSD was found in the 26 included studies all over the world (Fig. 5). Based on the randomized design, the overall prevalence for lower back was 40.1% (CI 95%: 32.2–48.0%).

#### Shoulder

**Fig. 6:**
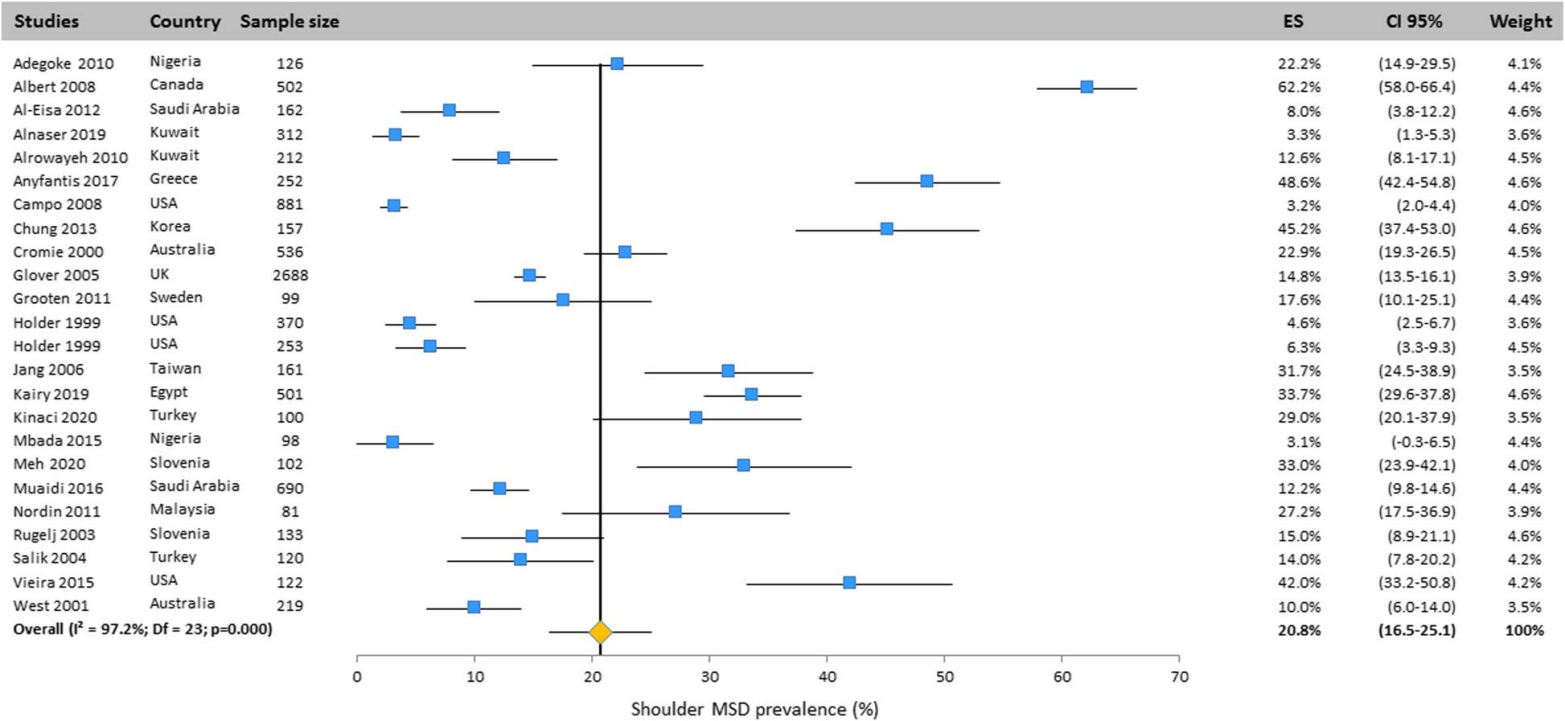
Prevalence of musculoskeletal disorders in shoulder amongst studies included

According to Fig. 6, the prevalence of shoulder MSD was mentioned in 24 studies. The results of the random effects model showed that the prevalence of this disorder was 20.8% (CI 95%: 16.5–25.1).

#### Elbow/forearm

**Fig. 7:**
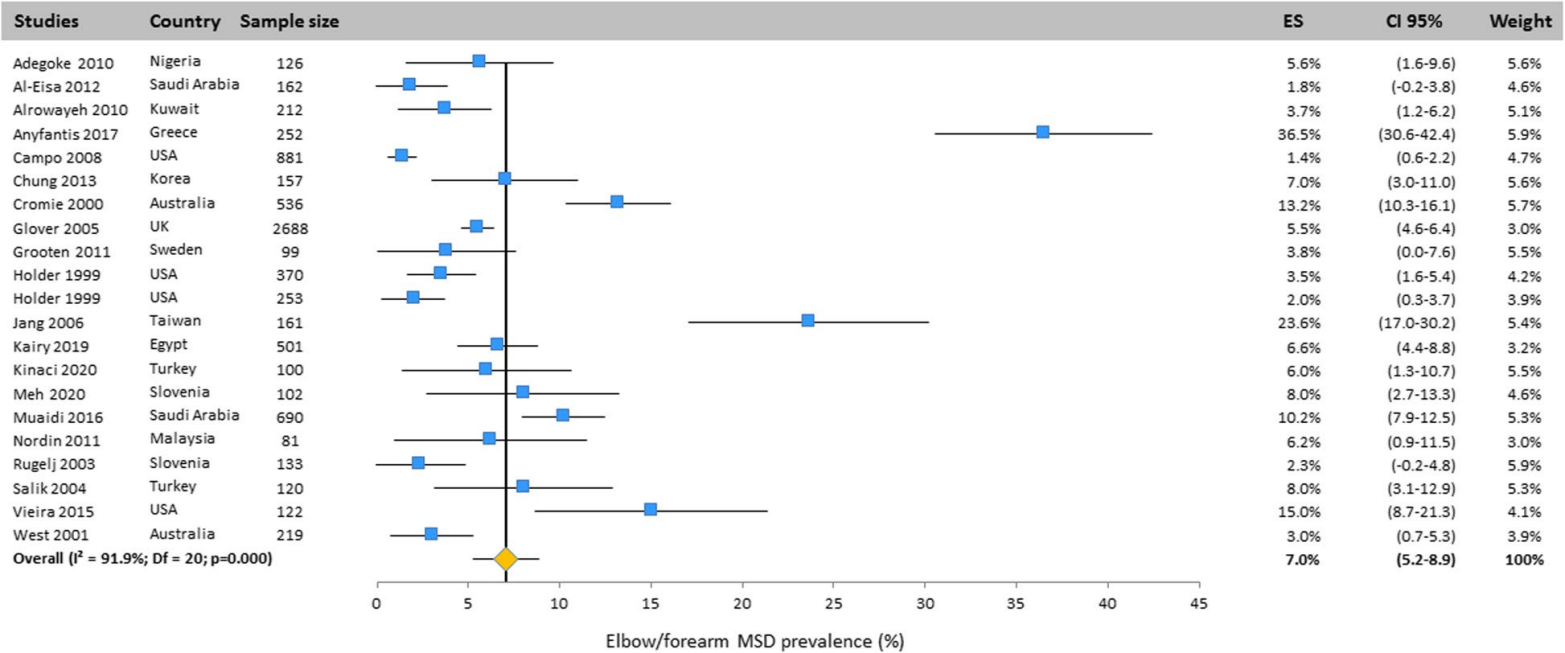
Prevalence of musculoskeletal disorders in elbow/forearm amongst studies included

The prevalence of elbow MSD has been presented in Fig. 7. This was assessed in 21 studies performed in many countries. Based on the results of the random effects model, its prevalence was 7.0% (CI 95%: 5.2–8.9).

#### Wrist/hand

**Fig. 8:**
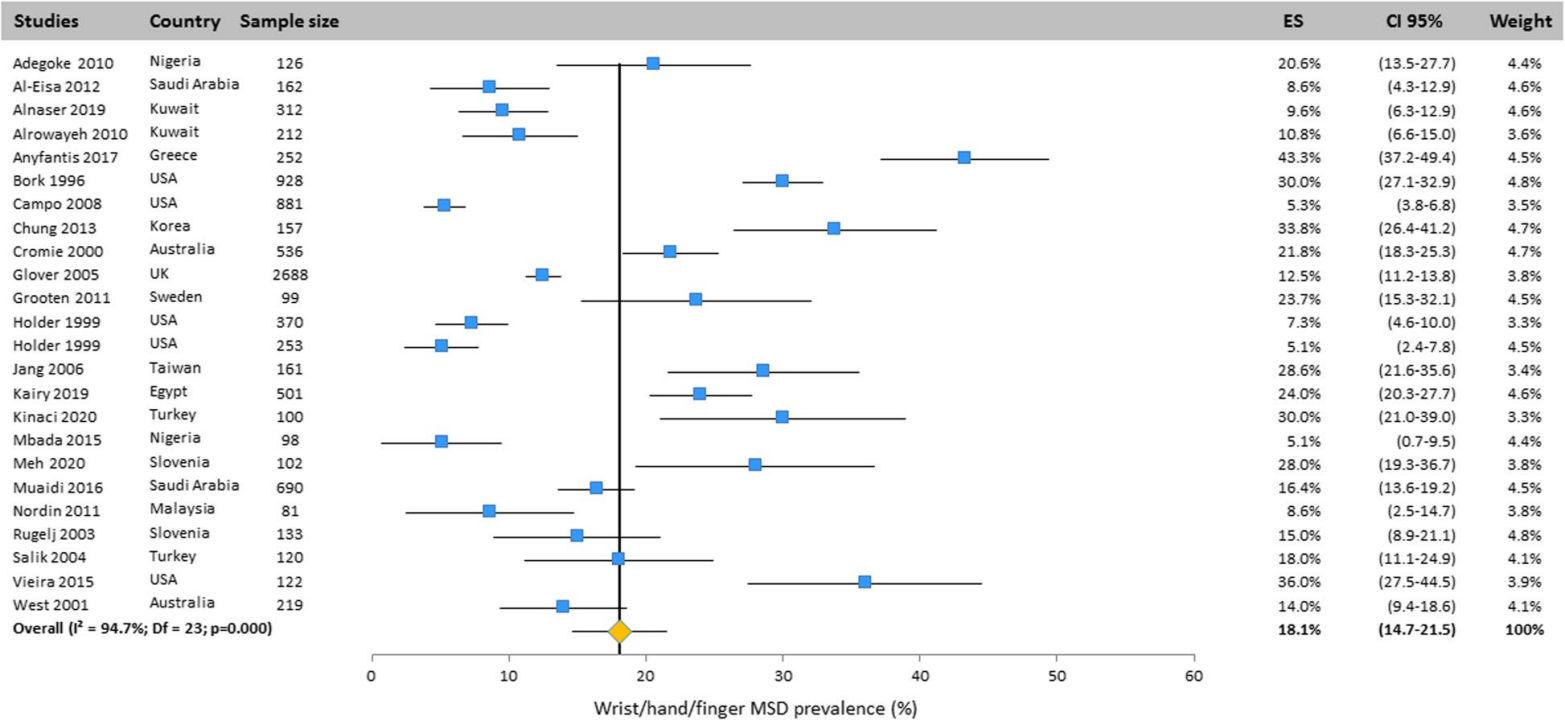
Prevalence of musculoskeletal disorders in wrist/hand amongst studies included

The wrist/hand MSD prevalence was evaluated in 24 studies spread over all the continents. The overall prevalence was 18.1% (CI 95%: 14.7–21.5%) obtained with the random effects model (Fig. 8).

#### Thumb

**Fig. 9:**
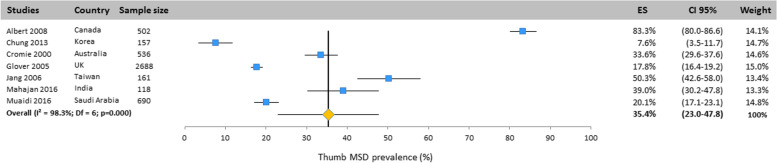
Prevalence of musculoskeletal disorders in thumb amongst studies included

The prevalence of MSD for the thumb was addressed in 7 studies conducted in Canada, Korea, Australia, the United Kingdom, the United States, Taiwan, India and Saudi Arabia (Fig. 9). Based on the results of the random effects model, the thumb MSD prevalence was 35.4% (CI 95%: 23.0–47.8).

#### Hip/thigh

**Fig. 10:**
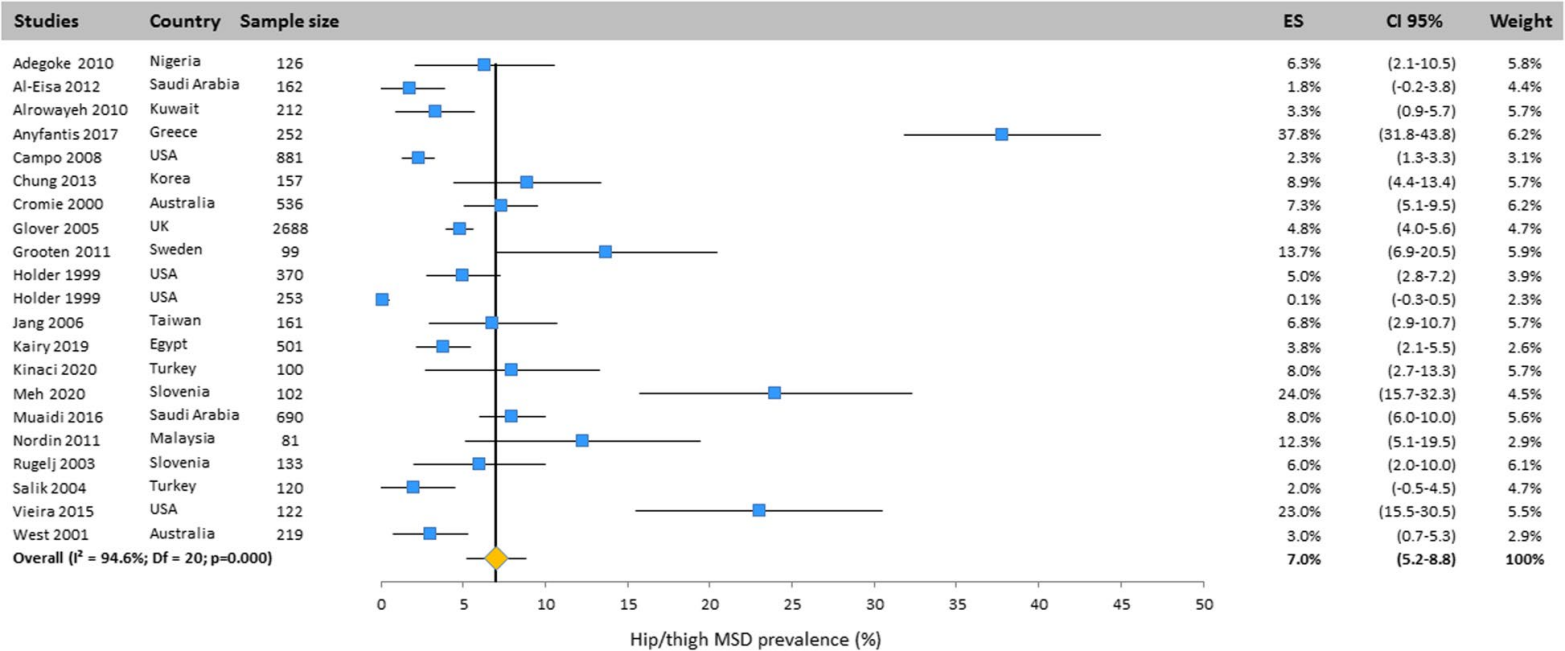
Prevalence of musculoskeletal disorders in hip/thigh amongst studies included

The prevalence of MSD in hip was reported in 21 studies conducted all around the world. The results of the random effects model showed that the prevalence of this disorder was 7.0% (CI 95%: 5.2–8.8) (Fig. 10).

#### Knee/leg

**Fig. 11:**
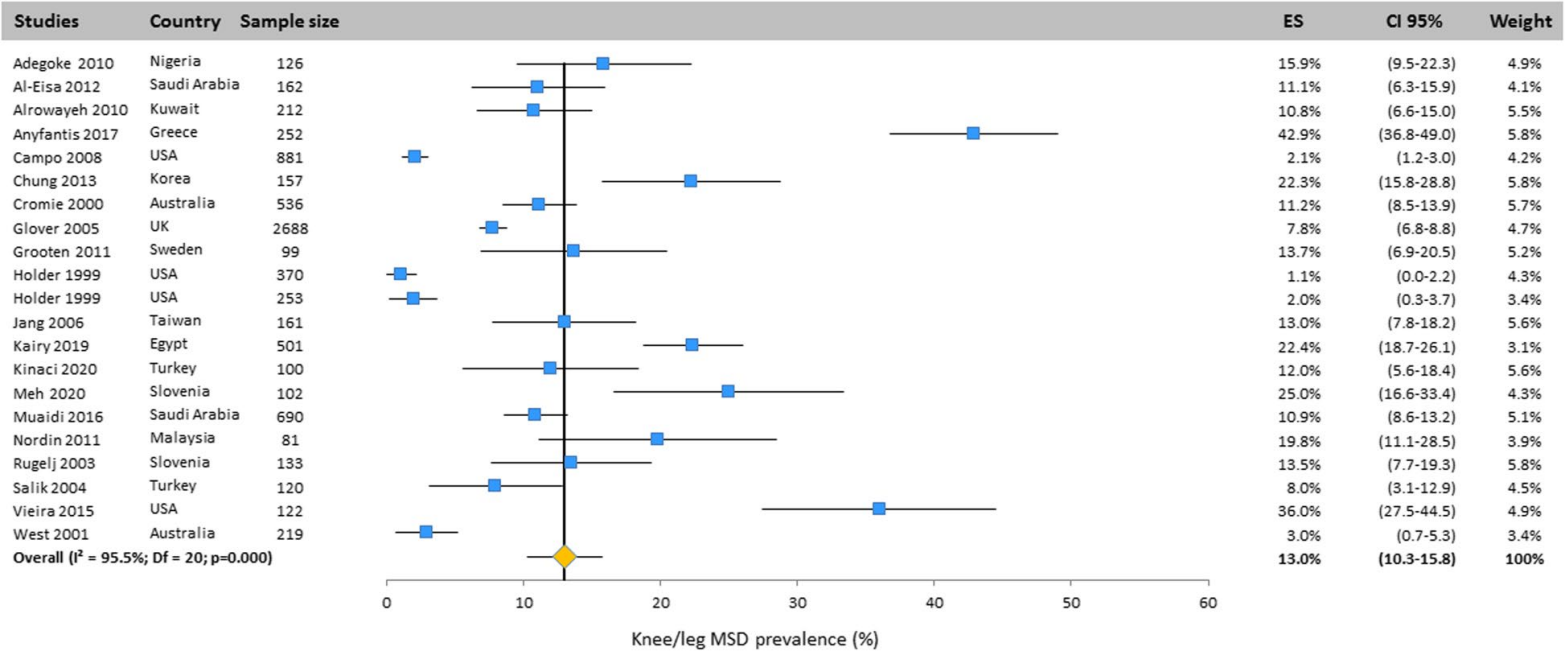
Prevalence of musculoskeletal disorders in knee/leg amongst studies included

The prevalence of knee/leg MSD has been presented in Fig. 11. This was assessed in 21 studies performed in many countries. Based on the results of the random effects model, its prevalence was 13.0% (CI 95%: 10.3–15.8).

#### Ankle/foot

**Fig. 12:**
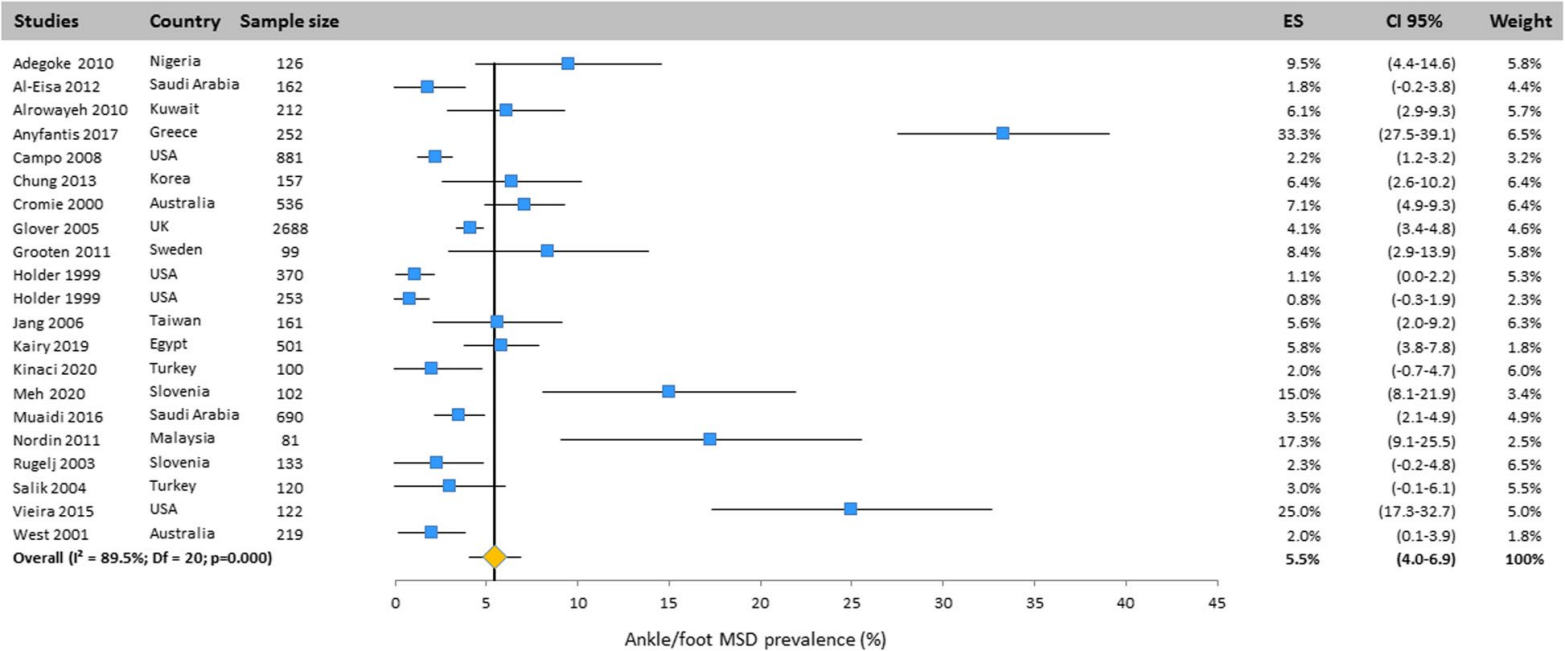
Prevalence of musculoskeletal disorders in ankle/foot amongst studies included

The prevalence of MSD in ankle/foot was also reported in 21 studies conducted all around the world. The results of the random effects model indicated that its prevalence was 5.5% (CI 95%: 4.0–6.9) (Fig. 12).

#### MSD prevalence by continent

Table 3 summarizes the sample size weighted mean prevalence of the eleven body areas by continent. About the lower back, the prevalence observed in Asia and Europe (36.7% and 41.0%) was close to the world value (40.1%). America presented a lower average rate (33.0%) while Africa and Oceania obtained higher prevalence (53.4% and 50.8% respectively).

**Table 3:** Sample size weighted average (standard deviation) of MSD prevalence by continent for each of the 11 body areas studied

		Neck	Upper back	Mid back	Lower back	Shoulder	Elbow	Wrist/ hand/ fingers	Thumb	Hip	Knee	Ankle/feet
Africa	Mean	26.8%	20.0%	-	53.4%	27.6%	6.4%	20.9%	-	4.3%	21.1%	6.5%
	SD	15.9%	11.5%	-	29.0%	15.5%	0.7%	10.1%	-	1.8%	4.6%	2.6%
America	Mean	23.4%	15.1%	-	33.0%	20.0%	3.0%	16.0%	83.3%	3.1%	4.4%	3.4%
	SD	28.8%	14.4%	-	25.4%	26.9%	6.4%	15.0%	-	11.1%	17.1%	11.8%
Asia	Mean	22.6%	9.5%	20.8%	36.7%	16.2%	9.0%	16.9%	24.7%	12.5%	12.5%	4.7%
	SD	9.9%	6.5%	10.9%	10.7%	13.6%	6.7%	10.0%	19.1%	3.7%	4.9%	5.0%
Europe	Mean	27.5%	20.8%	-	41.0%	18.1%	7.8%	15.8%	17.8%	8.3%	11.4%	6.7%
	SD	17.4%	20.1%	-	19.1%	14.8%	14.3%	12.3%	-	13.8%	14.0%	12.6%
Oceania	Mean	39.6%	41.0%	11.0%	50.8%	19.2%	10.2%	19.5%	33.6%	6.1%	8.8%	5.6%
	SD	19.5%	-	-	28.6%	9.1%	7.2%	5.5%	-	3.0%	5.8%	3.6%
**Overall**		**26.4%**	**17.7%**	**14.9%**	**40.1%**	**20.8%**	**7.0%**	**18.1%**	**35.4%**	**7.0%**	**13.0%**	**5.5%**
p (Kruskal–Wallis)		*p* = 0.93	*p *= 0.54	*p* = 1.0	*p* = 0.51	*p* = 0.83	*p* = 0.73	*p* = 0.84	*p* = 1.0	*p* = 0.31	*p* = 0.15	*p *= 0.36

For neck, the world prevalence was close to the value observed in Africa (26.8%) and Europe (27.5%). Values were slightly lower in America and Asia (23.4% and 22.6% respectively). Oceania displayed higher prevalence of 39.6%.

For shoulder, America, Europe and Oceania showed prevalence close to the world value (20.8%). The rate was higher in Africa (27.6%) and slightly lower in Asia.

Finally, the prevalence of thumb was different, with a smaller number of studies including this area. Oceania reported a prevalence equivalent to the world value of 35.4% but with only one study. The rate is lower in Asia (24.7%) with 4 studies and in Europe (17.8%) with one study. The prevalence was higher in America (83.3%) with only one study but that was mainly targeted to this area.

Kruskal–Wallis analysis revealed no difference in prevalence between continents for all body areas (Table 3).

### Meta-regression

**Fig. 13:**
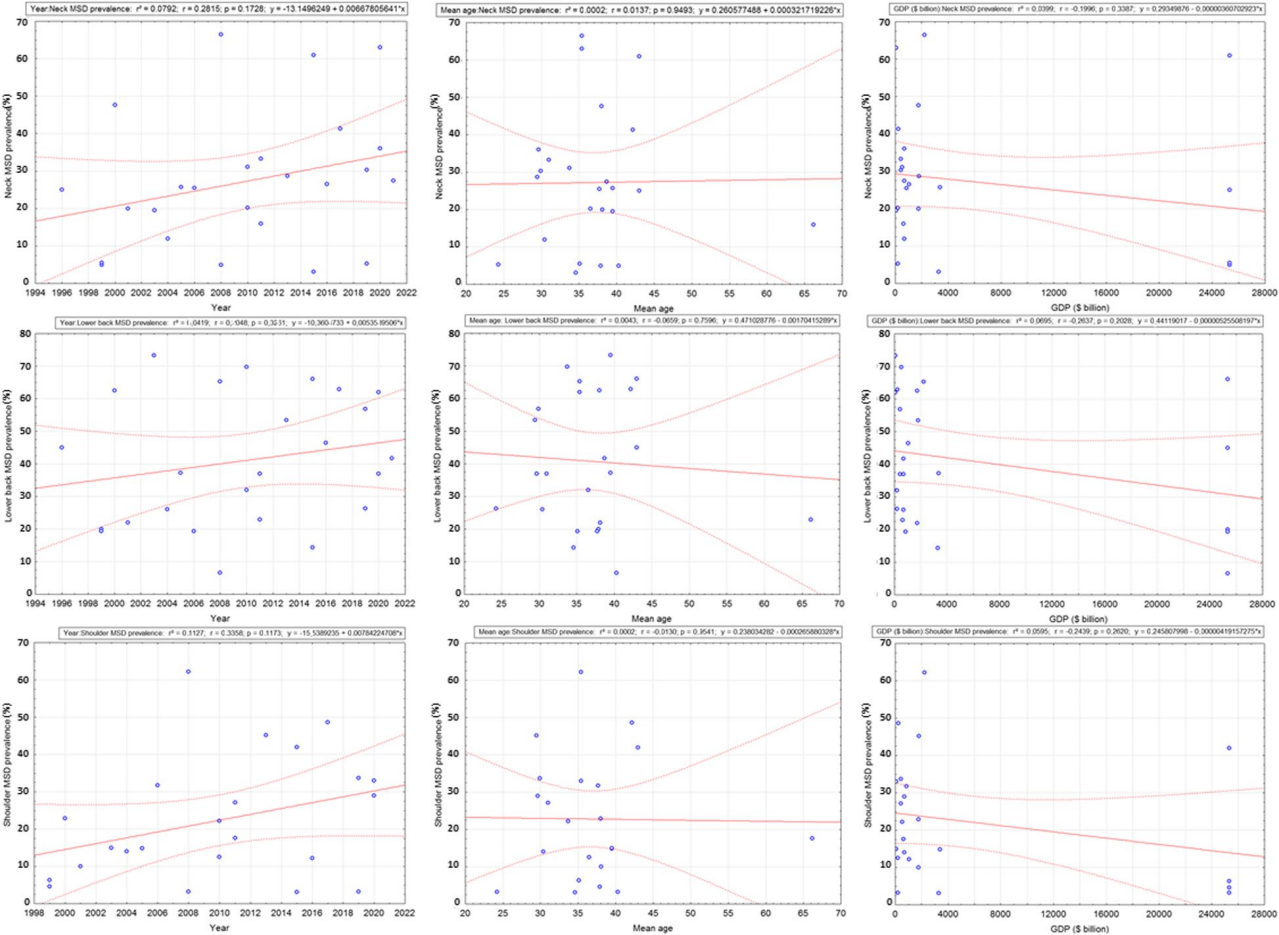
Meta-regression to evaluate the trends in the prevalence of neck, lower back and shoulder musculoskeletal disorders in relation to year of publication, age of participants and Gross Domestic Product (GDP)

Figure 13 illustrates the meta-regression performed for the neck, lower back and shoulder which were the most exposed areas to MSD. Whatever the body area, no effect of year of publication, means age of participants and GDP was evidenced (r^2^ between 0.0002 and 0.1127, *p* > 0.05).

## Discussion

The aim of this study was to propose a literature review and meta-analysis to investigate the prevalence of MSD among physiotherapists. The objective was to summarize the worldwide MSD prevalence by body area. Particular attention was paid to the way the results were presented in each study. In order to provide a synthesis that included comparable data, prevalence was recalculated when they did not refer to the global sample. The meta-analysis of the 26 included articles showed that the highest prevalence rates were observed for the lower back (40.1%), thumb (35.4%), neck (26.4%) and shoulder (20.8%). This global result was also observed in national studies. For example, for the lower back, the work of Glover et al. [6] in the UK (37.2%), Głowiński et al. [18] in Poland (41.7%) and [9] in Turkey (37.0%) reported equivalent rates. About the neck, several studies reported similar prevalence on all 5 continents: 30.3% in Egypt [8], 25.0% in the US [26], 26.5% in Saudi Arabia [7], 27.5% in Poland [18], and 20.0% in Australia [38]. For the shoulder, equivalent rates were observed mainly in Nigeria, Sweden and Australia with respective prevalence of 22.2% [16], 17.6% [10] and 22.9% [11]. The elbow and lower limbs were the areas least at risk for MSDs among physiotherapists (prevalence ranging from 5.5% for the ankle to 13% for the knee). Three studies presented prevalence 2 to 6 times higher than the others for the lower limb [17, 24, 29].

These results are consistent with the results proposed by Vieira in his review [2] or more recently in a study involving several health professionals including physiotherapists [39]. For all body areas, a large heterogeneity was observed between studies (I^2^ comprised between 76.4% and 98.3%). These levels are comparable to those observed in other meta-analyses carried out on other health professionals such as nurses (between 76.4% and 99.1% [40]) or surgeons (between 86.8% and 96.8% [4])Whatever the solutions, i.e. material, societal, related to working conditions or the work environment, it appears important to consider them to reduce MSD risks.

An interesting contribution of this meta-analysis was the consideration of MSD prevalence by continent. The aim was to find out whether there were differences between countries among physiotherapists. The analysis has shown that several results by continent were close to the world average (Table 3). For neck, four continents had prevalence differences of less than 4% with the global value of 26.4%. About the lower back, Asia and Europe showed prevalence close to the global values (40.1%) with respective rates of 36.7% and 41.0%. The other three continents had prevalence higher or lower by about 10%. For the shoulder, values similar to the world average (< 5% difference) were found for four continents. Only Africa had a higher rate of 7% (27.6%). Concerning the thumb, a great disparity of results was observed between continents. Only Oceania, through the study of Cromie et al. [11], presented values close to the world value (33.6% vs 35.4%). This can be explained by the few number of studies that specifically distinguished the MSD risks of the thumb from the rest of the hand, which was studied by the majority of studies (24/26). Despite the differences in prevalence observed, no significant difference was found between the continents. This could be explained by the high standard deviations obtained for each of the continents and more generally by the important heterogeneity of the data reported in the different studies included.

In order to have a world assessment of the MSD prevalence among physiotherapists, the search presented in this review was initiated with a reduced number of keywords. This led to the integration of works with very different methodological characteristics. Indeed, as shown in Table 2., the age of the participants varied between 20 and 65 years, which leads to differences in years of experience (0.5 to 40 years) and therefore in exposure to MSDs [10, 24, 25]. As shown by Molumphy et al. [41] for work-related lowed back pain in physical therapists, the age of the populations tested and consequently their professional experience may affect the responses to the questionnaires on the presence of MSD. Other studies, such as Grooten et al. [10] included only women, while other studies included a mixed population. In many studies, the work environments were not explicitly detailed. However, working in a public or private hospital can influence working conditions such as the total number of working hours, number of hours in direct contact with patients, equipment used, etc. These differences may affect the risk of MSDs or their perception. All these parameters characteristic of the samples tested could explain in part the high variability of MSD risks worldwide. The lack of covariance shown by the meta-regression between the parameters studied and the prevalence of MSDs explains reinforces this idea. It would therefore be relevant to take these different parameters into account as inclusion criteria in order to try to reduce the heterogeneity between studies and therefore the results. The question would then arise as to the relevance of a meta-analysis since there would probably be fewer studies included per criterion studied.

### Limitations

As mentions above, the main limitation was the heterogeneity. It was also observed into the questionnaires used to investigate MSD. Their nature could influence the results collected, particularly the fact that certain body area prevalence were not reported in several studies. On the other hand, some areas were presented differently in relation to the method used. For example the back could be divided into two (upper and lower back) or three (upper, mid and lower back) parts. Similarly, the thumb may or may not be included in the MSD assessment of the fingers of the hand.

This heterogeneity also affects the weight of the different studies in the meta-analysis. Indeed, for equivalent sample sizes, the method used increases the weight for studies with lower prevalence. However, a greater weight is given to studies with larger sample sizes.

To overcome these problems, it would be recommended to set up a more standardized protocol allowing all the information to be filled in homogeneously. Another alternative would be to pay more particular attention to the experimental conditions and characteristics of the populations in future reviews. This would allow considering only studies whose parameters would be quantified in a same experimental context and thus reduce heterogeneity. Indeed, different workplaces (public vs. private), gender, experience, age, etc.… are all factors that can affect the occurrence of MSDs. This would provide a more accurate and complete summary of the MSD prevalence among physiotherapists in the context of their professional activity. Psychosocial and societal factors that affect the occurrence of MSD should also be taken into account to complete the assessment. It would also be interesting to have a more precise idea of the practices carried out, particularly in terms of physical demands on the musculoskeletal system, to estimate the impact of the main activity on MSD risks. The work presented shows that increasing the number of studies would likely help reduce heterogeneity in future meta-analyses.

## Conclusion

The literature review, meta-analysis and meta-regression showed the presence of MSD with the highest worldwide prevalence located in the lower back, neck, shoulders and extremities of the hand independently of the continent. Methodological recommendations have been proposed to reduce the heterogeneity observed for future reviews and meta-analyses.

## Data Availability

We propose a literature review and a meta-analysis. All data are from the articles listed in the summary tables included in the manuscript.

## Abbreviations

MSD: Musculoskeletal Disorders
PT: Physiotherapist
PRISMA: Preferred Reporting Systematic Reviews and Meta-Analyses
CI: Confidence Intervals
